# Functional Nanostructures from Sol–Gel Synthesis Using Keggin Polyoxometallate Phosphotungstic Acid as a Precursor

**DOI:** 10.1021/acs.inorgchem.3c04122

**Published:** 2024-02-07

**Authors:** Björn Greijer, Wannes De Turck, Geoffrey Daniel, Jayeeta Saha, Mats Johnsson, Gulaim A. Seisenbaeva, Vadim Kessler

**Affiliations:** † Department of Molecular Sciences, Swedish University of Agricultural Sciences, Box 7015, Uppsala 75007, Sweden; ‡ Department of Forest Biomaterials and Technology, Swedish University of Agricultural Sciences, Box 756 51, Uppsala 75007, Sweden; § Department of Materials and Environmental Chemistry, Arrhenius Lab, Stockholm University, Stockholm 106 91, Sweden

## Abstract

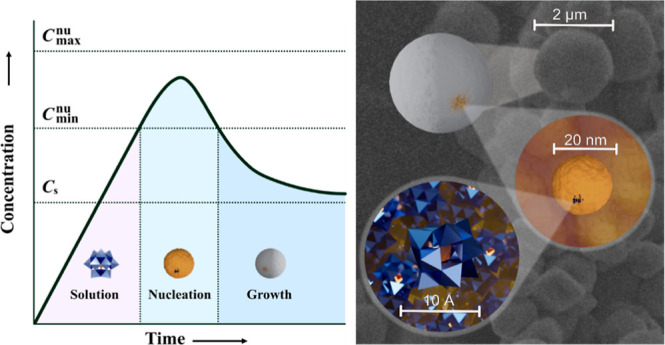

Subjecting phosphotungstic acid solutions to low pH in combination with introduction of polyvalent cations led to the formation of nanostructured microspheres of approximately 2 μm in size, as shown by scanning electron microscopy, which were almost insoluble and resistant to degradation at neutral and high pH. These microspheres were composed of secondary nanospheres with diameters around 20 nm as revealed by transmission electron microscopy and atomic force microscopy. Investigations of the crystal structure of a potential intermediate of this process, namely, acidic lanthanum phosphotungstate, [La(H_2_O)_9_](H_3_O)_3_[PW_12_O_40_]_2_(H_2_O)_19_, showed a tight network of hydrogen bonding, permitting closer packing of phosphotungstic acid anions, thereby confirming the mechanism of the observed self-assembly process. The new material demonstrated promising electrochemical properties in oxygen evolution reactions with the high stability of the obtained electrode material.

## Abstract 2

Directing self-assembly of the Keggin polyoxometalates permits to construct extremely stable hierarchical nanostructures showing activity in the electrochemical oxygen evolution reaction.

## Introduction

The internationally recognized goal to reduce evolution of greenhouse gases has set focus on the development of efficient fossil-free technologies for energy production.^1^ The main source of usable energy for the earth is solar light and thus solar energy conversion is one of the most addressed topics in modern research in designing and producing new materials.^2,3^ An attractive alternative to fossil fuel-based processes is hydrogen energy, primarily the production of electricity with the aid of fuel cells,^4^ but also the use of hydrogen in reduction reactions such as the recently proclaimed HYBRIT technology for “green synthesis” of steel from iron ore.^5^ A common feature of hydrogen energy technologies is the need for high-purity hydrogen gas required in large volumes. Its production is possible either via costly purification of hydrogen obtained via the water–gas shift reaction from natural gas or biogas^6^ or via highly energy-demanding electrolytic water splitting.^7^ An attractive alternative to electrolysis is the use of photocatalytic or electrocatalytic water decomposition. In these approaches, the energy costs for hydrogen gas can be significantly reduced while not compromising its quality. The challenge, however, lies in the need for expensive components used in the making of such catalysts. Typical photocatalysts for water splitting are nanoparticles (NPs) of semiconductor oxides or chalcogenides in combination with noble metals.^8^ The efficient electrocatalysts applied so far also commonly contain platinum group metal-based NPs either as oxides (RuO_2_^9^ or IrO_2_^10^) or together with platinum NPs.^11^ The development of noble metal free photo- and, especially, electrocatalysts is an important and highly addressed research target.^1^

An attractive candidate for the photo- and electrocatalyst in water splitting [i.e., oxygen evolution reaction (OER)] is pure^12−16^ and chemically doped^17−19^ tungsten oxide along with closely related nanocomposite heterostructures.^20−23^ The challenge in the creation of such related nanostructures is the relatively high reactivity and solubility of WO_3_ and its derivatives in both acidic and basic media.^24,25^ Here, in particular, phosphotungstic acid has attracted attention as a possible photocatalyst^26,27^ and potentially electrocatalyst^21^. However, this compound in its hydrated form is highly soluble in water, which hinders its application.

Phosphotungstic acid is a well-studied representative of the polyoxometallate (POM) family of compounds. It is of the Keggin type, the smallest metal oxide nanoparticle of approximately 1 nm in diameter, which has a highly ordered structure consisting of a central heteroatom inside a cage of 12 transition metal atoms in their highest oxidation state, all connected by oxygen bridges, giving the general formula XM_12_O_40_^z–^ (X = P, Si, Ge, As, Sb; M = W, Mo; z = 3 or 4).

We have previously reported complexes of Keggin POMs and oligopeptides, in an effort to model the interactions between NPs and proteins at the nanoscale.^28,29^ Some of these complexes displayed POM–POM contacts, approximately 3 Å in length, likely resulting from hydrogen bonds. As these were observed at very low pH, protonation of the POM likely shielded the charge, allowing for direct interactions. Acidic conditions are necessary when working with Keggin POMs, as one drawback of phosphotungstic acid, in particular, is its instability at neutral and alkaline pH.^24,25^ Thus, retaining POM intact at a higher pH could potentially strongly expand its suitability as a catalyst.

**Figure 1:**
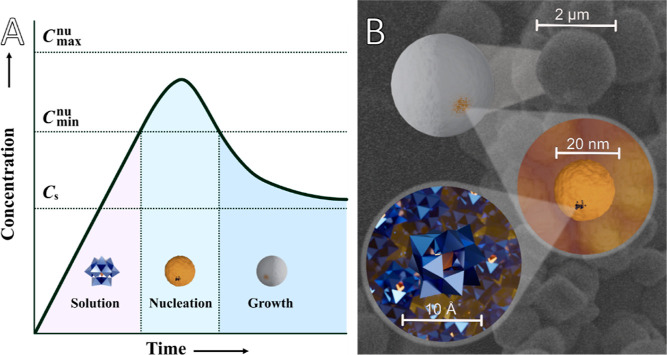
Hierarchical assembly of phosphotungstate spheres by the sol–gel process, illustrated in relation to the La Mer concept graphically (A) and structurally (B). Individual POMs aggregate to form nanoparticles approximately 20 nm in diameter, which in turn assemble into ternary particles of up to approximately 2 μm in diameter.

In the present study, we utilized the Keggin POM as a precursor in a sol–gel process. The sol–gel is a process where separation of a solid phase, usually metal oxide, from solution occurs via nucleation in the form of NP species resembling POMs. This creates a colloid solution (i.e., sol), allowing subsequent aggregation without growth, forming a colloid solid – gel.^30^ The Keggin POM species appear to act as such NPs and self-assemble into secondary particles, analogous to self-assembled particles formed by a sol–gel. The process can be described through a La Mer diagram,^31^ illustrated by a graph in Figure 1A. The conditions required for their formation in this case appear to be highly acidic media, heat, and the presence of polyvalent cationic species in addition to the POM opening for neutralization of the POM charge and subsequent aggregation. Using this approach, we were even able to isolate and characterize a chemically individual intermediate in this self-assembly process—a lanthanum salt of POM. The produced hierarchical self-assembled POM structures were extremely poorly soluble under acidic and neutral conditions and demonstrated exceptional stable activity in electrochemical water splitting.

## Results and Discussion

In a recent study, we found that increasing acidity and ionic strength (cation concentration) in solutions of Keggin POMs together with peptides resulted in the formation of compounds with lower peptide-to-POM ratios, where metal cations and, most importantly, oxonium ions became incorporated into the resulting structures.^32^ Highly charged cations facilitated formation of “acidic” POM derivatives that are usually included in the composition of the product. In the present study, we attempted to use extremely acidic conditions with pH < 0 and relatively high concentrations of highly charged cations such as Ti(IV), Zr(IV), Ce(IV), and La(III) on heating with continuous stirring. As expected, this approach resulted in all cases in hierarchical self-assembly of POMs with formation of spherical aggregates several micrometers in size. The analysis of the particles showed a hierarchical structure with three levels of organization (Figure 1). First, the POM “nuclei” make contact at similar distances via hydrogen bonds. Second, nanospheres made from POMs of approximately 20 nm in diameter are formed. Third, assemblies of these nanospheres form ternary particles of up to 2 μm in diameter.

**Figure 2:**
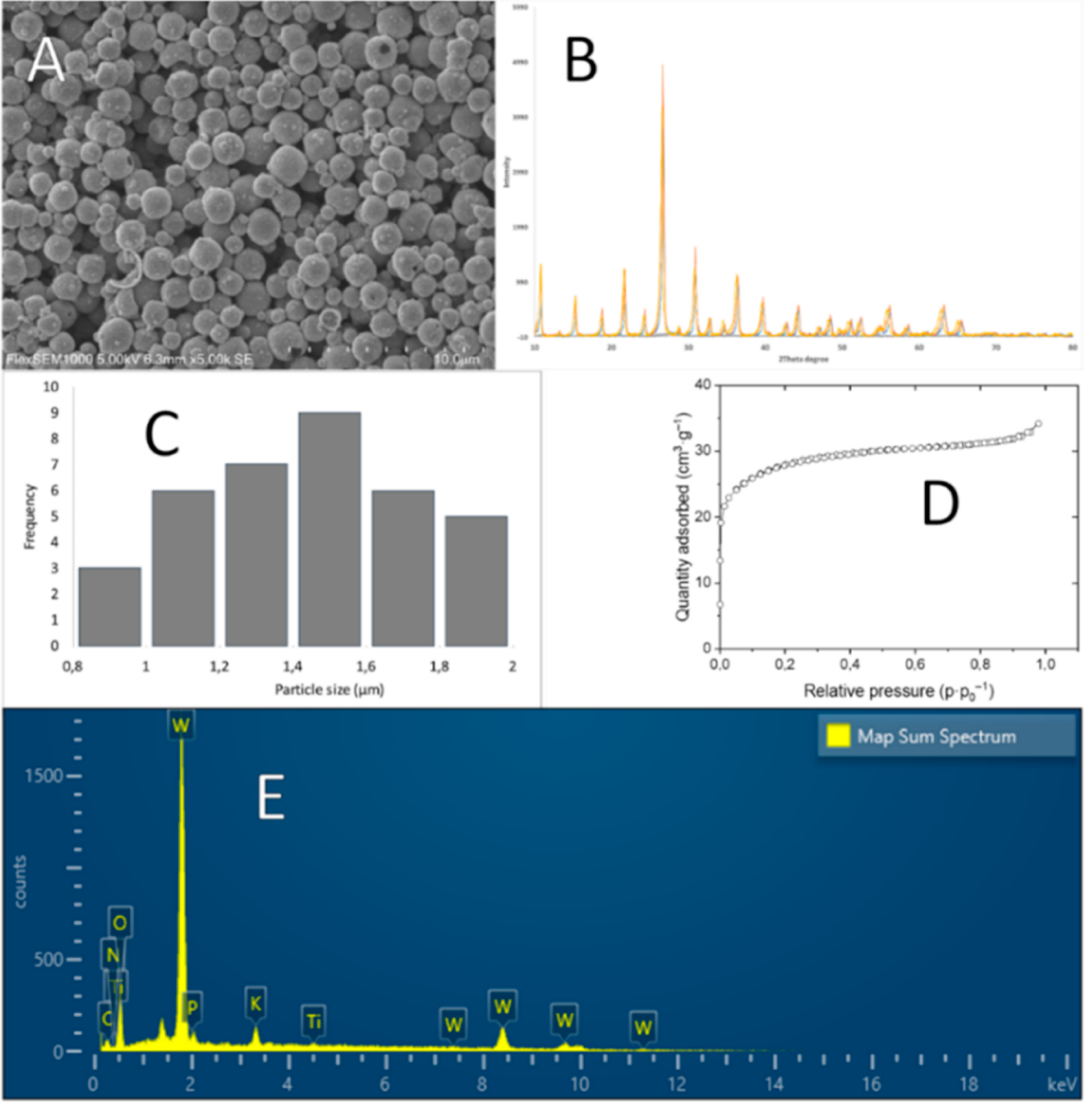
(A) SEM image of the Ti-derived spheres at 5000× magnification. (B) XRPD pattern of the spheres. (C) Size distribution of the particles in A ranges from 0.8 to 2 μm, with an average of 1.4 μm. (D) Nitrogen adsorption/desorption isotherms for PW NPs. (E) EDS spectrum of the spheres. Tungsten and oxygen were the most abundant, while traces of potassium and titanium were also detected.

Scanning electron microscopy (SEM) images revealed spherical particles ranging from 500 nm to 2 μm in diameter (Figure 2A). A typical size distribution for Ti(IV)-derived material (i.e., nanospheres/particles) is shown in Figure 2C. Debris of broken spheres were present in the unwashed samples. EDS analysis showed mainly tungsten, with traces of the metal cations present during synthesis, such as titanium and potassium (Figure 2E and Table TS1).

X-ray powder diffraction (XRPD) of freshly obtained materials was consistent with the known pattern of hydrated Keggin-type phosphotungstic acid H_3_PW_12_O_40_·21H_2_O (Figure 2B).^33^ Drying of the samples resulted in broadening of the peaks and weakening of the peak intensity, with a fully X-ray amorphous product on longer storage. These transformations were likely caused by the loss of water molecules from the material that became amorphous while preserving the overall morphology at all levels.

The nitrogen adsorption/desorption isotherms for the PW NP sample are shown in Figure 2D. The shape of the isotherms corresponds to characteristic type I, typical of microporous solids having relatively small external surfaces, the limiting uptake being governed by the accessible micropore volume rather than by the internal surface area.^34^ The Brunauer–Emmett–Teller specific surface area (*S*_BET_) was found as 100.6 m^2^·g^–1^ and the Langmuir surface area was found as −125.9 m^2^·g^–1^ for the PW NP sample. The Barrett–Joyner–Halenda desorption cumulative surface area and volume of pores between 1.7 and 300 nm were 23.0 m^2^·g^–1^ and 0.019 cm^3^·g^–1^ (corresponding to 8.75 vol % of pores), respectively.

**Figure 3:**
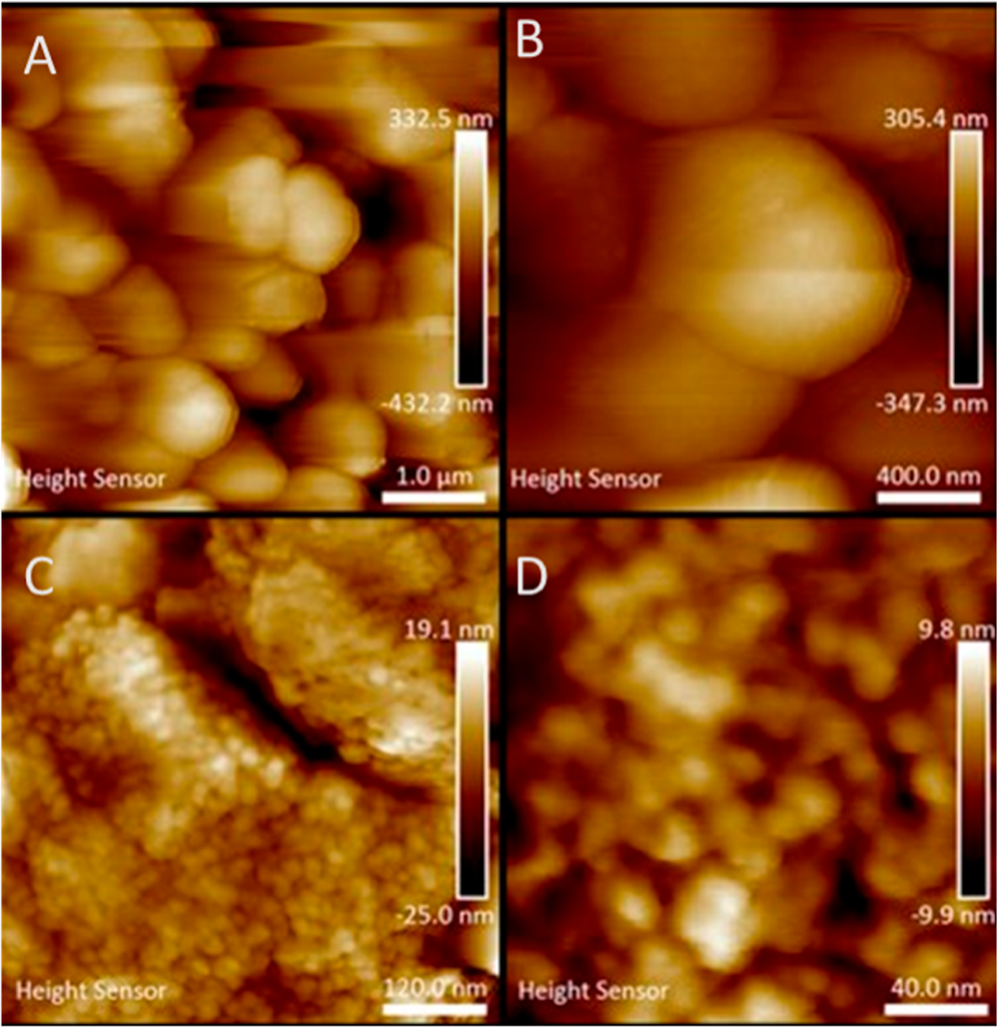
AFM images of the spheres. Particles in the micrometer range (A,B) can be seen at low magnification and their nanosized composition at high magnification (C,D).

Atomic force microscopy (AFM) investigations showed that the spheres were made up of fairly uniform secondary particles (Figure 3), although larger than individual POMs, which are primary ones in this sol–gel process, analogues of what in metal–organic sol–gel are called micelles templated by the self-assembly of ligands (MTSALs).^35,36^ This implies, at the first step, aggregation of the Keggin POMs in the tens of nm size, which then form a tertiary aggregate in the μm range. The secondary particles, as shown from the X-ray diffraction (XRD) data, were originally formed as crystallites of the H_3_PW_12_O_40_·21H_2_O phase. Their growth, however, is apparently impeded by adsorption of highly charged cations on their surface, which drastically decreases solubility of the material and permits aggregation of these secondary particles.

**Figure 4:**
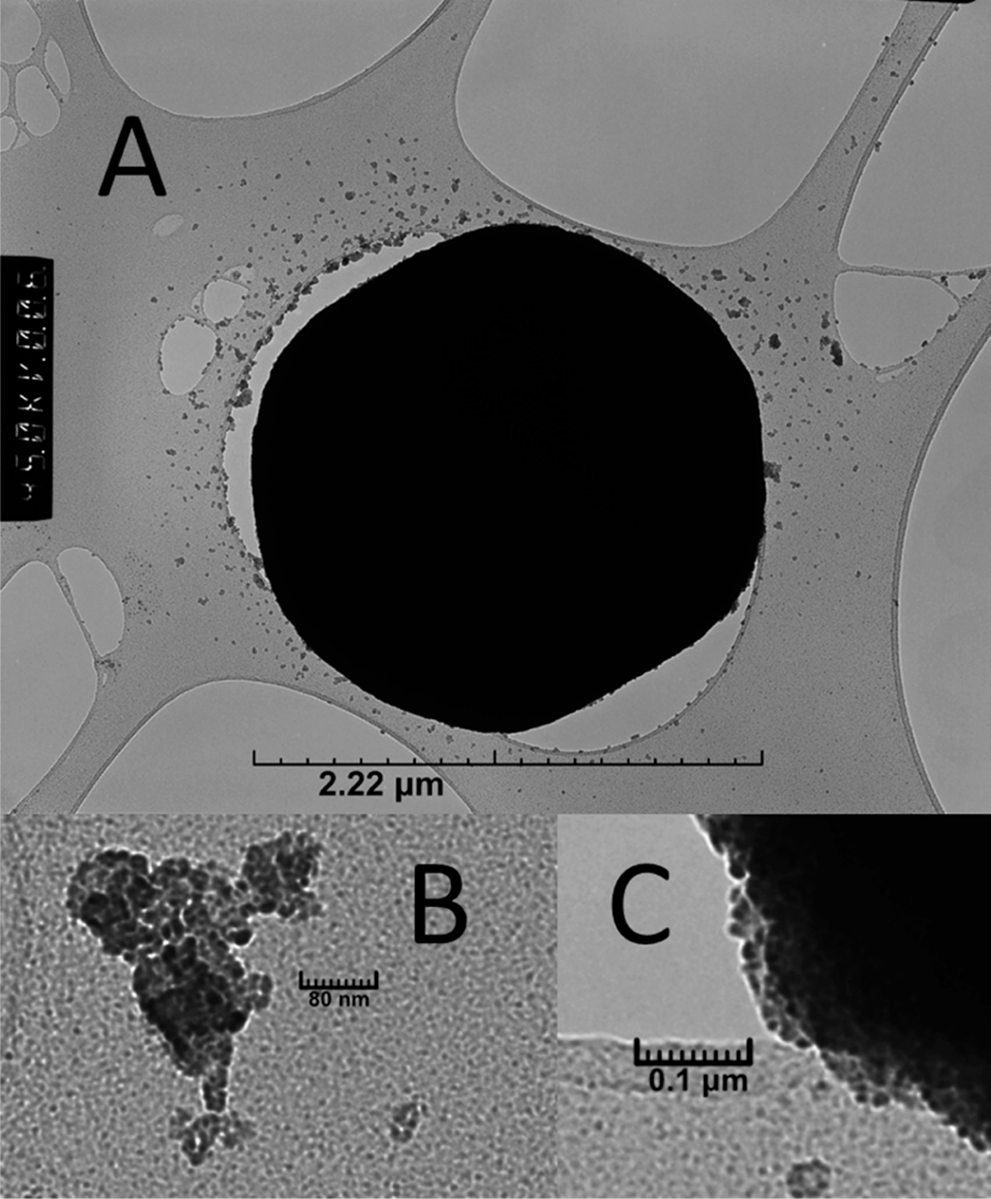
TEM image of the spheres. The large tertiary particle (A) is made up of secondary particles of nanosize (B,C).

The hierarchical composition of the spheres was also observed under transmission electron microscopy (TEM), where tertiary spheres of approximately 2 μm across (Figure 4A) are made up of secondary spheres in the tens of nanometers in diameter (Figure 4B,C) that are presumably composed of individual units of hydrated phosphotungstic acid.

Similar structures, made of potassium phosphotungstate, have been reported by Yan et al.,^26,37^ although these were produced by simple coprecipitation through dropwise addition of KCl solution to a POM solution. The latter were significantly smaller and singular in composition and structure rather than the hierarchical structure observed in this work. It can be speculated that they were formed by the action of analogous driving forces with their ground in charge compensation of the POM structural units. The cation content and the solubility, crucial for the application as stable electrocatalysts, are, however, drastically lower in the new material reported here.

The thermal stability of the produced material was investigated via both thermogravimetric analysis (TGA) (Figure S1) and by preparative experiments. The weight loss occurred in several steps in the interval 120–500 °C, being associated initially with the loss of the different forms of water content. Elemental analysis of the residues showed that the ratio of W to P did not vary significantly in the samples taken at different temperatures (σ = 0.78), and thus, no loss of the phosphorus content could be observed.

For dynamic light scattering (DLS) and zeta potential, three measurements were taken using distilled water as a medium, and the mean and standard deviation are shown in Supporting Information Table TS2. As the particles were fairly large, the standard deviation of the zeta potential was near 5 in all cases.

### Single-Crystal X-ray Structure of the Isolated Intermediate

**Figure 5:**
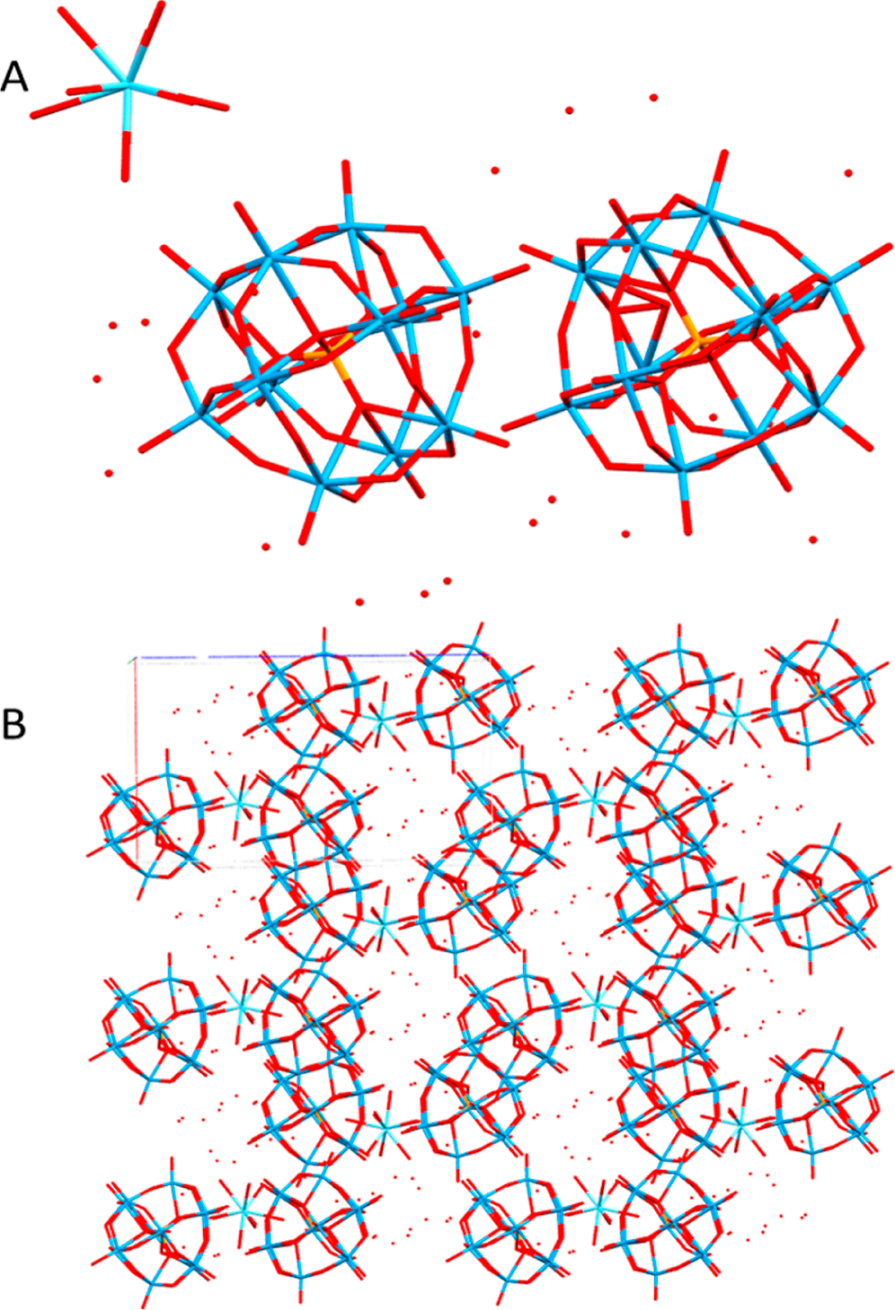
Asymmetric unit (A) and packing (B) of the phosphotungstic acid crystals of acidic lanthanum phosphotungstate, [La(H_2_O)_9_](H_3_O)_3_[PW_12_O_40_]_2_(H_2_O)_19_. Numerous cavities filled with water molecules are seen as well as close contacts between POMs.

Crystals isolated from the synthesis using La(III) nitrate were triclinic centrosymmetric with a *P*1̅ space group, containing two phosphotungstate anions, one lanthanum ion, and 31 water molecules in an asymmetric unit, *Z* = 2 (Figure 5, Table 1). The composition of the material can thus be formulated as [La(H_2_O)_9_](H_3_O)_3_[PW_12_O_40_]_2_(H_2_O)_19_. The large amount of water forms an extensive hydrogen bonding network throughout the crystal. The shortest contacts lie between water at 2.16 and 2.7 Å. At a bond length of 2.7–2.8 Å, contacts exist between both water molecules and water to bridge POM oxygen. Between 2.8 and 2.9 Å, there are a number of bonds mainly between water and between water and terminal POM oxygen. In this range, there are also contacts between water and bridging POM oxygen, as well as POM–POM contacts. The longest contacts above 2.9 Å lie between water, POM oxygen and water, or two adjacent POMs.

The extensive contacts between POMs suggest that they are protonated at this pH (Figure S2), allowing for hydrogen bonding between the POMs. Though the protons are not visible in the structure, the bond distances are consistent with H-bonds. Similar phenomena have been observed with phosphomolybdic acid previously.^29^ The water molecules participate mostly in four hydrogen bonds per molecule, which is typical for the structure of liquid water. We can observe two POMs per La ion, necessitating the need for other cations (e.g., protons) to contribute to charge neutralization. This structure can be seen as a “snapshot” of an intermediate in the process by which the spheres form, the next step being the accumulation of a larger number of POM units and transfer of the cations to the surface of the hydrated phosphotungstic acid crystallites.

**Table 1:** Details of Data Collection and Refinement for the Phosphotungstate–Lanthanum Structure^a^

chemical formula	H_65_LaO_113.50_P_2_W_24_
formula weight	6494.77 g/mol
temperature	163(2) K
wavelength	0.71073 Å
crystal size	0.160 × 0.200 × 0.320 mm
crystal system	triclinic
space group	*P*1̅
unit cell dimensions	*a* = 14.008(3) Å α = 88.978(5)°
	*b* = 15.140(3) Å β = 89.228(4)°
	*c* = 22.386(5) Å γ = 80.885(5)°
volume	4686.7(18) Å^3^
*Z*	2
theta range for data collection	2.26 to 28.00°
index ranges	–18 ≤ *h* ≤ 18, −19 ≤ *k* ≤ 19, −29 ≤ *l* ≤ 29
reflections collected	57 188
Independent reflections	22 324 [*R*(int) = 0.0652]
No. of observed independent reflections; *I* > 2σ(*I*)	17 582
final *R* indices, observed	*R*1 = 0.0596, w*R*2 = 0.1531
final *R* indices, all data	*R*1 = 0.0787, w*R*2 = 0.1630

a Details of data collection and refinement can be obtained free of charge from the Cambridge Crystal Structure Database at https://www.ccdc.cam.ac.uk/structures/citing, deposition no. CSD-2307589.

### Electrochemistry

**Figure 6:**
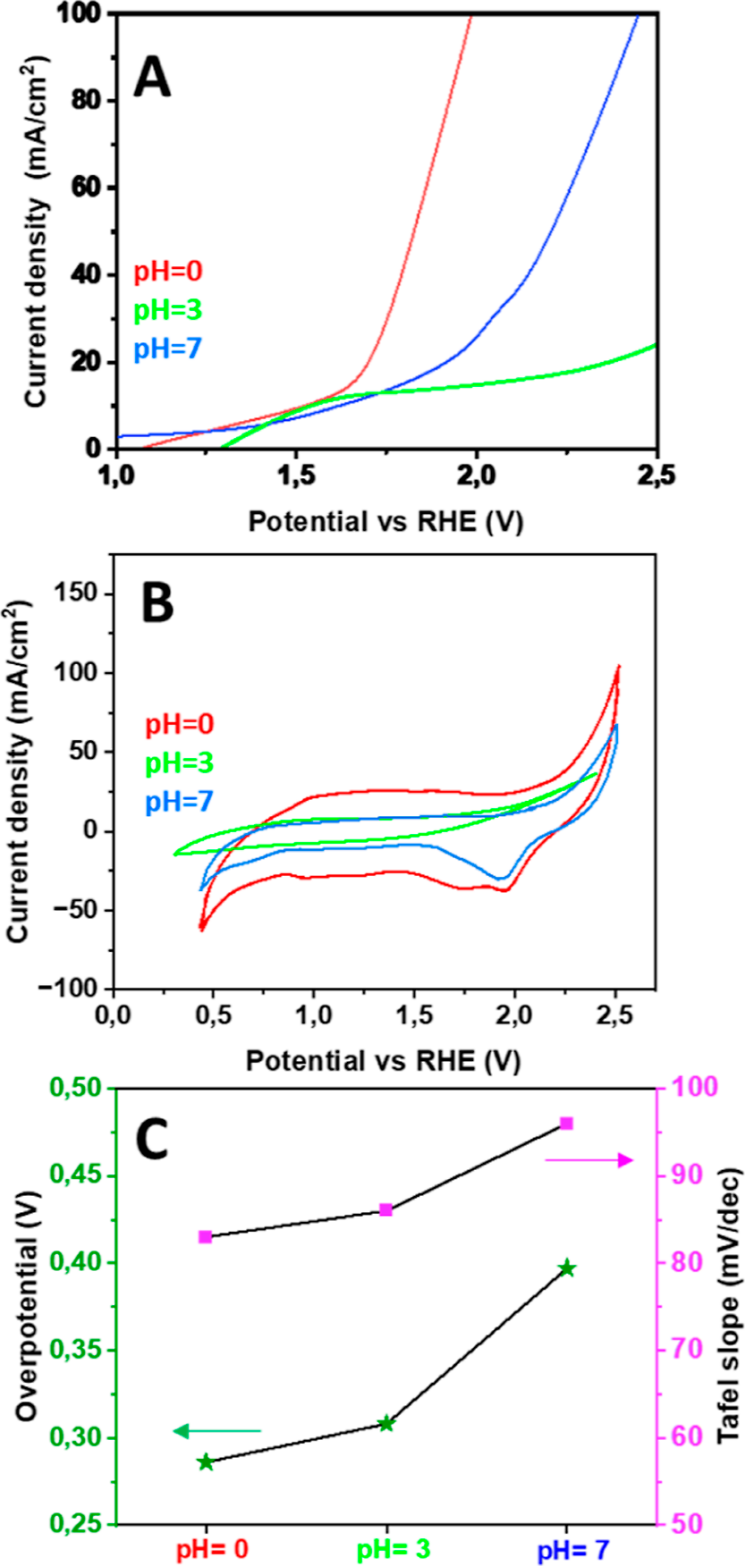
Electrochemical measurements at pH 0, 3, and 7, where phosphotungstic acid has been evaluated as an electrocatalyst for the OER in water splitting. (A) LSV. (B) CV curves obtained after initial stabilization. (C) pH dependence of the overpotential and Tafel slope.

Phosphotungstic acid has been used as an electrocatalyst at different pH values from 0 to 7 for the OER in water splitting. The catalyst is highly dependent on the pH of the electrolyte and LSV measurements give the lowest overpotential at acidic conditions, see Figure 6. At pH 0 (0.5 M H_2_SO_4_), the overpotential was as low as 286 mV for the formation of *O*_2_(*g*) bubbles (OER). At pH 3 (citric acid/sodium citrate buffer), the overpotential was 308 mV and at pH 7 (phosphate buffer), the catalyst showed an overpotential of 397 mV. The formation of oxygen bubbles becomes more and more pronounced with increasing potential. The determined faradaic efficiency was quite high, exceeding 90% and slightly increasing in time (see Supporting Information Table TS3–5).

From the cyclic voltammograms obtained at pH = 7, a redox peak was observed at 0.72 V, confirming the W^6+^/W^5+^ redox state, while at pH = 0, two redox peaks were observed at 0.72 and 0.50 V, confirming both W^6+^/W^5+^ and W^5+^/W^4+^ redox states, respectively.^15^ The shift in the redox peak confirms the influence of phosphorus on the redox potential of tungsten, as phosphorus incorporation enhanced the acidity.^19^

The Tafel slopes calculated from the linear sweep voltammetry (LSV) measurements showed the efficiency of an electrode to produce current in response to a change in the applied potential. The Tafel slope increases with pH in the tested range: 83 mV/dec at pH 0, 86 mV/dec at pH 3, and 96 mV/dec at pH 7. Chronoamperometry (CA) was performed to evaluate the stability of the catalyst over time. The current density was observed during 24 h and found to be stable at an applied potential of 0.4 V (vs RHE) (Figures S3–S5 and Table S3).

## Conclusions

Exploiting the factors leading to a decrease in the potential surface charge of phosphotungstic acid nanocrystals allowed the development of nanostructured microspheres of this material by simple sol–gel synthesis. Determination of the crystal structure of the potential intermediate product—an acidic salt of La(III) cations—provided additional clues to how the self-assembly process occurs. Sol–gel-produced water-insoluble phosphotungstic acid was demonstrated as a potential candidate for an electrocatalyst for the OER process at acidic conditions showing a very low overpotential in 0.5 M H_2_SO_4_ (pH = 0) electrolyte with great stability.

### Experimental Section

All chemicals were purchased from Sigma-Aldrich and used without further purification. The particles were produced by the general procedure of using 30 mL of a solution containing 1 mM of the cation Ti(IV) from potassium titanium oxide oxalate hydrate, or La(III) from lanthanum nitrate with pH near 0 (<0.1). To this was added 1 mM of phosphotungstic acid, upon which a precipitate formed. The precipitate was filtered either immediately or after the solution was evaporated to a near volume of 10 mL in a water bath held above 90 °C. The precipitate was washed with Milli-Q water and collected by centrifugation. Crystals of La and phosphotungstic acid were prepared by dissolving 0.4 g of lanthanum nitrate in 30 mL 2 M HCl and heating the solution in a > 95 °C water bath. To this was added 3 g of phosphotungstic acid and the solution was evaporated to approximately 12 mL, without stirring, at which point large cube-shaped crystals formed. Upon cooling, small X-ray quality crystals formed. The crystals were stable under the mother liquor, but upon drying, they degraded into a white powder. ***Safety concern***: using relatively concentrated acidic solutions at near boiling water temperature is associated with a risk of stench of highly corrosive and irritating liquid—the use of gloves and safety goggles is compulsory on operation.

SEM and energy-dispersive X-ray spectroscopy (EDS) samples were immobilized on carbon tape and characterized using a Hitachi FlexSEM-1000 II. EDS spectra were analyzed using an Oxford Instruments EDS analysis system operated by the Aztec software.

For TEM observations, dispersions of the sol–gel were deposited on holey carbon grids (PELCO 50 mesh grids: pitch 508 μm; hole width 425 μm; bar width 83 μm; transmission 70%) and observed using a Philips CM/12 microscope (Thermo Fisher Inc.) fitted with a LaB_6_ gun and operated at 100 kV. Negative TEM films were scanned using an Epson Perfection Pro 750 film scanner.

BET specific surface area and pore volume were determined from nitrogen adsorption/desorption isotherms at −196 °C (Micromeritics ASAP 2020 surface area and porosity analyzer). The samples were degassed at 120 °C for 12 h before measurements.

DLS and zeta potential were done by suspending spheres in distilled water and analyzing them on a Malvern Panalytical Zetasizer Nano analyzer, equipped with a red (362.8 nm) laser. Data was processed using the Zetasizer Ver. 7.12 software.

For AFM, samples were characterized using a Bruker Dimension FastScan atomic force microscope with a Nanoscope V controller in the ScanAsyst mode using a FastScan-B AFM probe (silicon tip, f_0_:400 kHz, k:4 N/m, tip radius: 5 nm nominally) and a scan rate of 1–3 Hz. Data was processed using Bruker NanoScope Analysis.

Preparative TGA was performed using a Nobertherm LE/6/11/P300 furnace and an FA2204B electronic balance. Approximately 160 mg of PW spheres was placed in two crucibles and heated to 120, 170, 220, 270, 320, 400, and 500 °C. At each point, the weight of one crucible was recorded, and from the other, a sample was taken for the EDS analysis.

Single-crystal XRD data was collected on a Bruker D8 QUEST ECO instrument and processed using the Apex4 software. A total of 2424 frames were collected. The total exposure time was 2.02 h. The frames were integrated with the Bruker SAINT software package using a narrow-frame algorithm. The integration of the data using a triclinic unit cell yielded a total of 57 188 reflections to a maximum θ angle of 28.00° (0.76 Å resolution), of which 22 324 were independent (average redundancy 2.562, completeness = 98.7%, *R*_int_ = 6.52%, *R*_sig_ = 8.17%) and 17 582 (78.76%) were greater than 2σ(*F*^2^). The final cell constants of *a* = 14.008(3) Å, *b* = 15.140(3) Å, *c* = 22.386(5) Å, α = 88.978(5)°, β = 89.228(4)°, γ = 80.885(5)°, and volume = 4686.7(18) Å^3^ are based upon the refinement of the *XYZ*-centroids of 9879 reflections above 20 σ(*I*) with 4.512 < 2θ < 71.58. Data were corrected for absorption effects using the multiscan method (SADABS). The ratio of minimum to maximum apparent transmission was 0.307. The calculated minimum and maximum transmission coefficients (based on the crystal size) were 0.0370 and 0.0870. The structure was solved and refined using the Bruker SHELXTL software package, using space group *P*1̅, with *Z* = 2 for the formula unit, H_65_LaO_113.50_P_2_W_24_. The structure loses water extremely easily, which likely creates multiple defects reflected in low precision in determination of the electron density, in spite of using low-temperature data collection. This generated a B-alert for large residual electron density. The B-alerts for isolated oxygen atoms are actually misleading because these atoms are actually water molecules invoked into a network of hydrogen bonding. The location of hydrogen atoms was impossible to discern because of the challenges in obtaining the correct electron density map. The final anisotropic full-matrix least-squares refinement on *F*^2^ with 1268 variables converged at *R*1 = 5.96% for the observed data and w*R*2 = 16.30% for all data. The goodness-of-fit was 0.990. The largest peak in the final difference electron density synthesis was 9.579 e^–^/Å^3^ and the largest hole was −6.835 e^–^/Å^3^ with an RMS deviation of 0.749 e^–^/Å^3^. On the basis of the final model, the calculated density was 4.588 g/cm^3^ and *F*(000), 5631 e^–^. The full list of bond distances and angles is available in Supporting Information Tables S7–S9.

All electrochemical experiments were performed at room temperature. The experiments were performed in a three-electrode system using an SP-50 potentiostat (Biologic). The phosphotungstic material was tested as an electrocatalyst at different pH from pH 0–7 for the OER in water splitting. The catalyst was then mixed with carbon black to enhance the conductivity and subsequently deposited on the graphite felt (loading = 0.2 mg/cm^2^). A three-electrode setup was used to perform the electrocatalysis experiments; a catalyst-loaded graphite working electrode, Pt-mesh as a counter electrode, and Ag/AgCl as a reference electrode. CV, LSV, and CA were performed. OER tests were carried out in a single-compartment electrolytic cell with different electrolytes of 0.5 M H_2_SO_4_ (pH = 0), citric acid/sodium citrate buffer (pH = 3), and phosphate buffer (pH = 7). For cyclability, 200 cycles of CV were performed, and the working electrode saturated after 20–30 cycles of activation. The *iR* drop was directly compensated for by the potentiostat (with 82% compensation). The potentials recorded were finally calibrated in relation to the reversible hydrogen electrode (*E*_RHE_) by using the equation: *E*_RHE_ = *E*_Ag/AgCl_ + 0.059 × pH. To minimize the capacitive current, the scan rate for the LSV curve was 10 mV/s. The overpotential (η) of HER was calculated by using the equation: η = *E*_RHE_ – 1.23, after reduction of the redox potential of oxygen, *E*_O2/O2–_ = 1.23. The Tafel plots were obtained by transforming the LSV curve into log(*j*) vs *E*. All experiments were performed twice to check reproducibility. The faradaic efficiency was evaluated via control of the gas evolution. The instrumental setup and procedure details are reported in the Supporting Information (Figure S7 and Tables S3–S5).

## Supporting Information Available

Additional experimental data; graphs and tables for TGA, EDS, DLS, and electrochemical measurements; and crystallographic data (PDF)
