# Supplementary material for: Functional Nanostructures from Sol–Gel Synthesis Using Keggin Polyoxometallate Phosphotungstic Acid as a Precursor
Source: Inorg Chem. 2024 Feb 7;63(7):3428–35. doi: 10.1021/acs.inorgchem.3c04122 (PMC10880052; doi:10.1021/acs.inorgchem.3c04122)
Supplement: Supplementary file 1 — ic3c04122_si_001.pdf [file ic3c04122_si_001.pdf]

Supporting information

Functional nanostructures from sol-gel synthesis using Keggin POM phosphotungstic acid as precursor

Björn Greijer,[⊥] Wannes De Turck,[⊥] Geoffrey Daniel,[] Jayeeta Saha,[ǂ] Mats Johnsson,[ǂ] Gulaim A. Seisenbaeva,[⊥] Vadim Kessler[⊥]*

[⊥] Department of Molecular Sciences, Swedish University of Agricultural Sciences, Box 7015, 75007 Uppsala, Sweden; [] Department of Forest Biomaterials and Technology, Swedish University of Agricultural Sciences, Box 756 51, Uppsala, Sweden; [ǂ] Department of Materials and Environmental Chemistry, Arrhenius Lab, Stockholm University, 106 91 Stockholm, Sweden

*vadim.kessler@slu.se

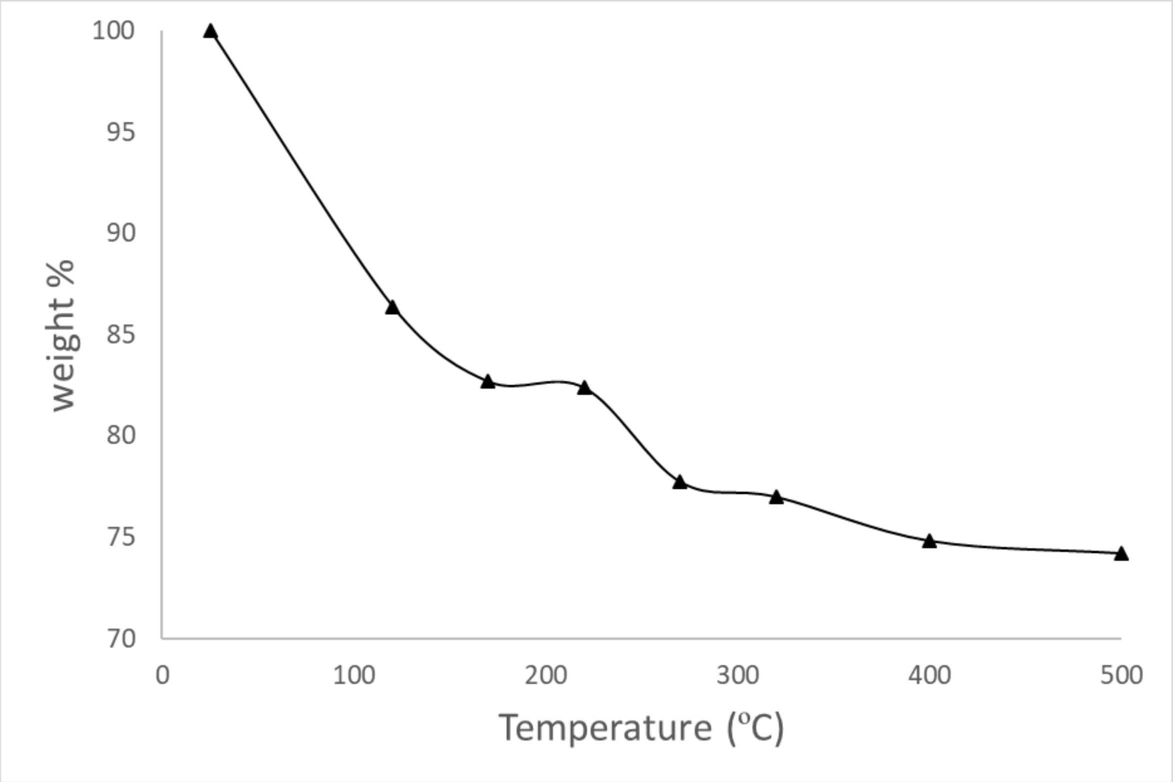

Figure S1. TGA curve of the spheres. A stepwise loss of water was observed.

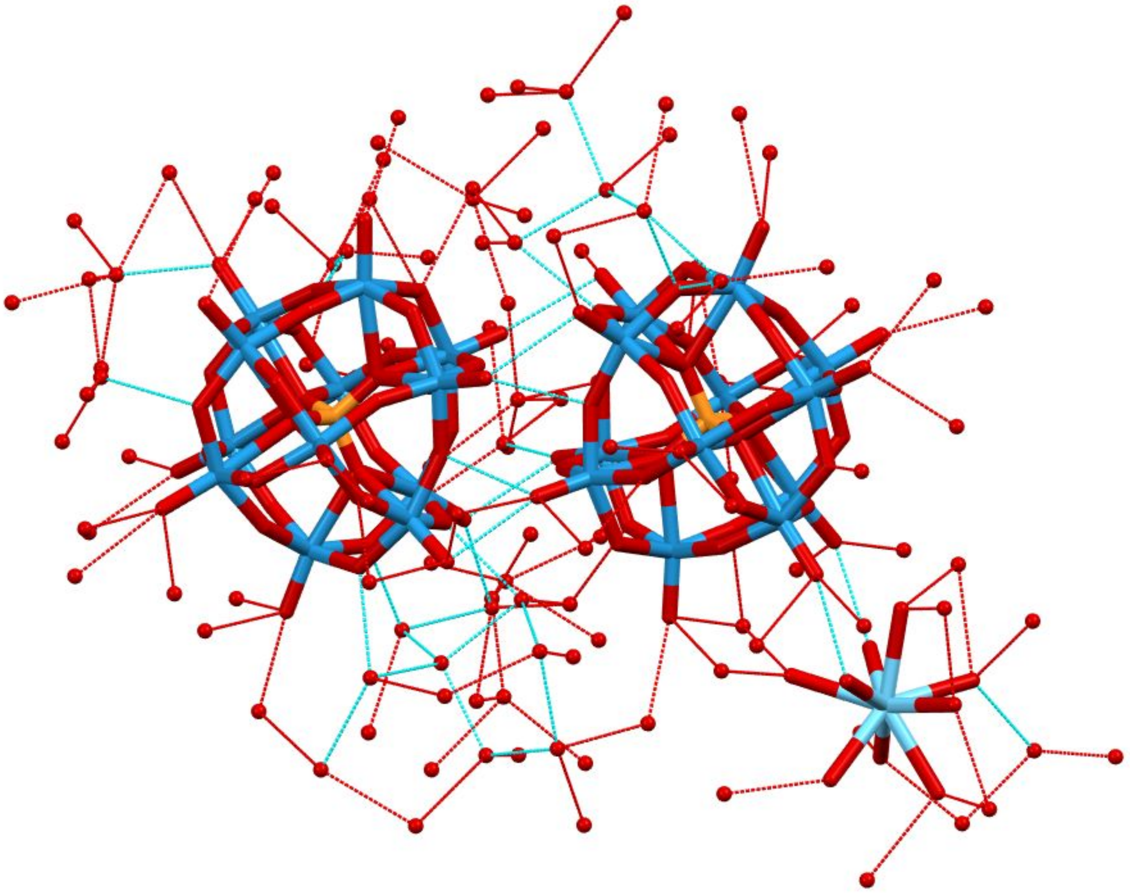

Figure S2. The scheme of hydrogen bonding in the structure of $[La(H_2O)_9](H_3O)_3[PW_{12}O_{40}]_2(H_2O)_{19}$.

Table TS1. EDS data of the spheres produced using Ti(IV) species.

**Map Sum Spectrum**				
**Element**	**Line Type**	**Weight %**	**Weight % Sigma**	**Atomic %***
W	M series	75.15	0.79	22.88
O	K series	20.03	0.75	70.07
K	K series	3.35	0.23	4.80
P	K series	0.83	0.26	1.51
Ti	K series	0.63	0.24	0.74
Total		100.00		100.00

* Tungsten and oxygen are most abundant, while traces of potassium and titanium can be detected. Phosphorus content is in agreement with that required for the Keggin POM composition.

Table TS2. DLS intensity and zeta potential mean and standard deviation

Mean Intensity (nm)	Mean Zeta potential (mV)
741,7 ± 324.6	-52,7 ± 5.58

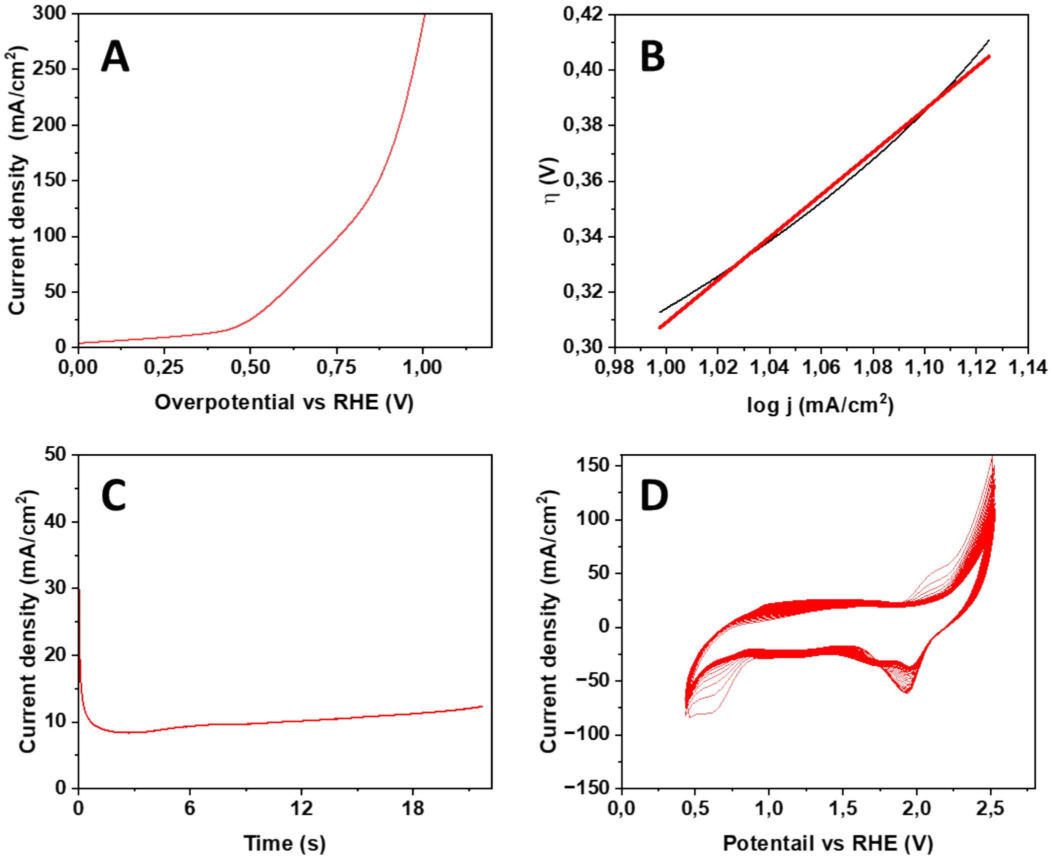

Figure S3. Electrochemical measurements at pH=0 (0,5 M H_2SO_4). (A) Linear sweep voltammetry (LSV), (B) Tafel slope -83mV/dec, (C) Chronoamperometry (CA), (D) Cyclic voltammetry (CV) stabilisation during 200 initial cycles.

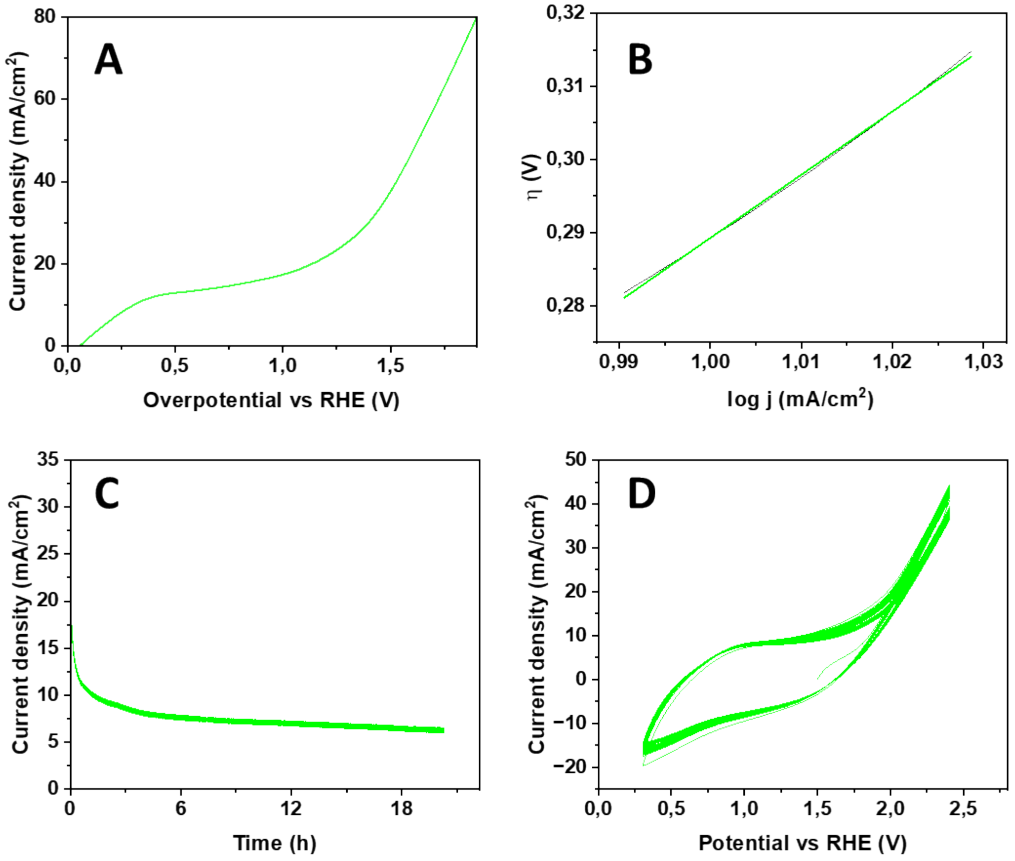

Figure S4. Electrochemical measurements at pH=3 (citrate buffer). (A) Linear sweep voltammetry (LSV), (B) Tafel slope -86 mV/dec, (C) Chronoamperometry (CA), (D) Cyclic voltammetry (CV) stabilisation during 200 initial cycles.

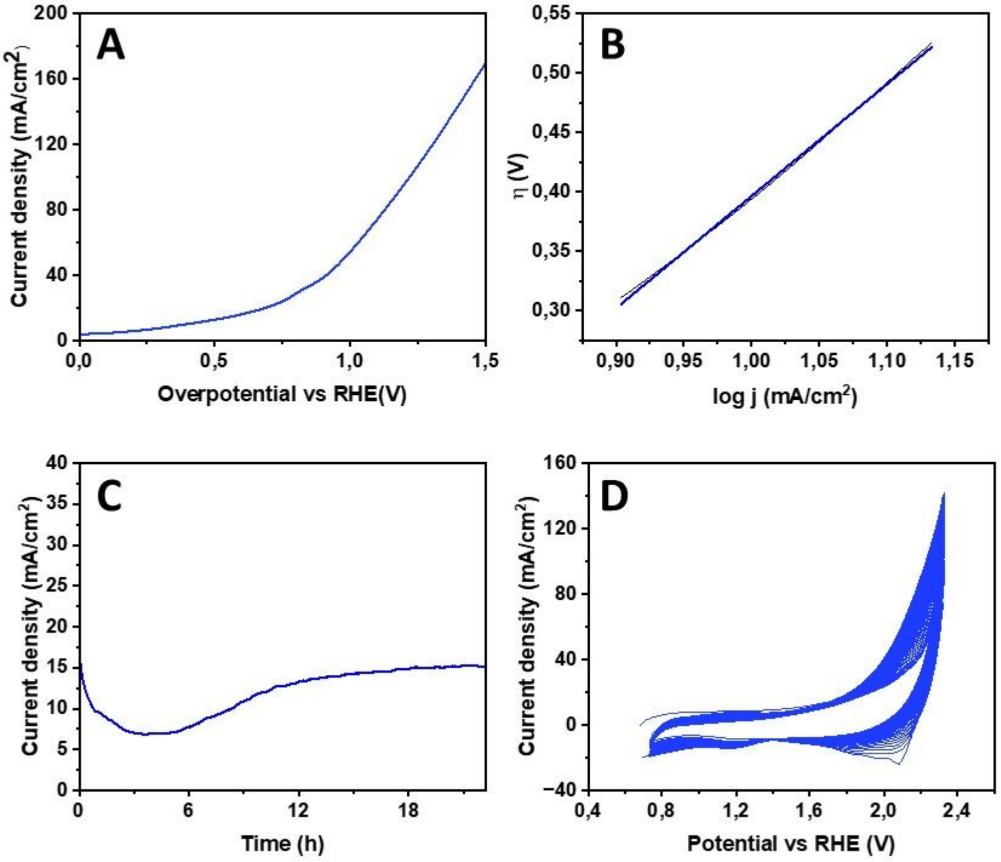

Figure S5. Electrochemical measurements at pH=7 (phosphate buffer). (A) Linear sweep voltammetry (LSV), (B) Tafel slope -86 mV/dec, (C) Chronoamperometry (CA), (D) Cyclic voltammetry (CV) stabilisation during 200 initial cycles.

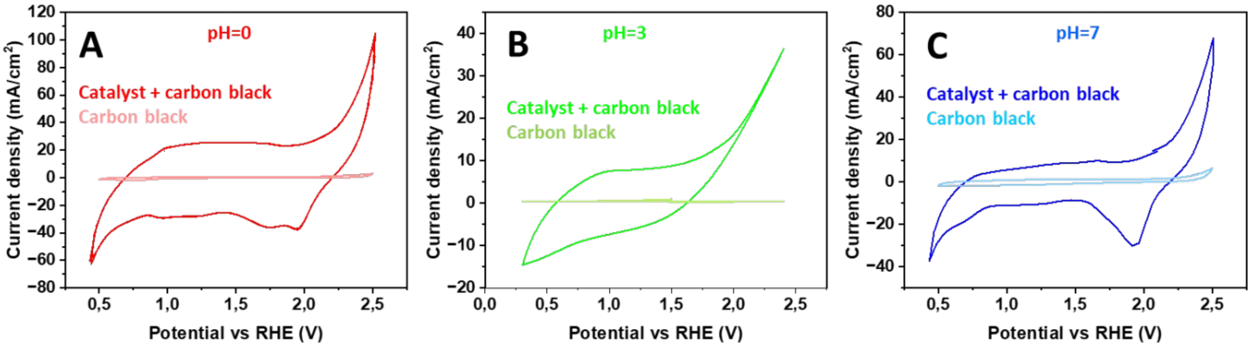

Figure S6. Cyclic voltammetry (CV) at pH 0 (A), 3 (B) and 7 (C) with phosphotungstic acid (catalyst) and carbon black. To demonstrate the OER activity of the catalyst, cyclic voltammetry was performed with catalyst combined with carbon black and pure carbon black deposited on the graphite electrode at different pH values of the electrolytes. We have observed that the carbon black shows no activity towards OER in any of the electrolytes tested, confirming the actual catalyst agent for the OER process is the phosphotungstic acid.

Faradaic efficiency

The Faradaic efficiency was calculated by collecting O_2(g) and H_2(g) using the water displacement method. One third of the total volume of gas should then be O_2(g) and two thirds H_2(g); $2H_2O = 2H_2 + O_2$.

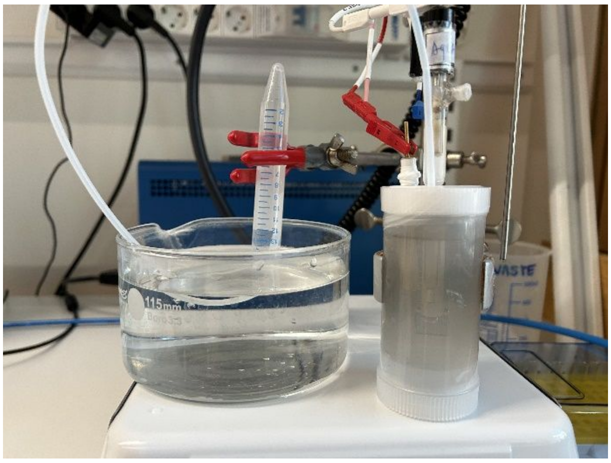

Figure S7. Gas evolution measurement setup

Table TS3. Chronopotentiometry at constant current of 10 mA for 30 min, 60 min, 90 min, 120 min respectively.

**Time (min)**	**30**	**60**	**90**	**120**
Total gas measured (mL)	5.7	11.7	17.7	24.0
H_2 (mL) calculated	3.8	7.8	11.8	16.0
O_2 (mL) calculated	1.9	3.9	5.9	8.0
O_2 (mg) calculated from measured volume **(m_{expt})**	2.7	5.6	8.42	11.42

Faraday's law of electrolysis, $m=\frac{Q \times M}{F \times z}$

Where, m=mass of oxygen in gram, Q=total charge passed through in Coulomb, F=96500 C/mol, M=molar mass of oxygen in g/mol, z= number of electrons transferred.

Applying the Faraday's law, theoretical mass of O_2 produced.

Table TS4. Mass of oxygen generated

Time (min)	30	60	90	120
O_2 (mg) **(m_{cal})**	2.98	5.96	8.94	11.92

Table TS5. Faradic efficiency: $\mathbf{m_{expt}/m_{cal} \times 100\%}$

Time (min)	30	60	90	120
Faradic efficiency (%)	91	93.5	94.2	95.8

Table TS6. All electrochemical measurements were made as double tests.

Catalyst	Electrolyte	pH	Overpotenti al (V)	Tafel slope (mV/dec)	Stability
$H_3PW_{12}O_{40}$	0,5 M H_2SO_4	0	0.286, 0.299	83, 76	24 h, 200 cy
$H_3PW_{12}O_{40}$	Citrate buffer	3	0.308, 0.314	86, 87	24 h, 200 cy
$H_3PW_{12}O_{40}$	Phosphate buffer	7	0.397, 0.434	96, 97	24 h, 200 cy

Table TS7. Atomic coordinates and equivalent isotropic atomic displacement parameters (Å2) for LaPW_LT12_221118.

U(eq) is defined as one third of the trace of the orthogonalized Uij tensor.

x/a y/b z/c U(eq)

W1	0.29675(4)	0.51497(4)	0.49322(2)	0.00657(11)
P1	0.28858(4)	0.28174(4)	0.49030(2)	0.010000
W2	0.38324(4)	0.38040(4)	0.36155(2)	0.00694(12)
W3	0.12260(4)	0.42470(4)	0.39807(2)	0.00722(12)
W4	0.11910(4)	0.19932(4)	0.39816(2)	0.00723(9)
W5	0.03501(4)	0.32399(4)	0.51952(2)	0.00723(9)
W6	0.44353(4)	0.13299(4)	0.58395(2)	0.00743(12)
W7	0.53809(4)	0.24154(4)	0.45115(2)	0.00794(12)
W8	0.28438(4)	0.04729(4)	0.49599(2)	0.00730(12)
W9	0.20107(4)	0.17278(4)	0.61700(2)	0.00732(12)
W10	0.20704(4)	0.41606(4)	0.61532(2)	0.00794(12)
W11	0.44912(4)	0.37563(4)	0.58290(2)	0.00703(12)
W12	0.38002(4)	0.15570(4)	0.36332(2)	0.00719(12)
W13	0.05702(4)	0.31088(4)	0.09331(3)	0.01142(13)
W14	0.15341(4)	0.41696(4)	0.96029(2)	0.00968(12)
W15	0.15466(4)	0.17321(4)	0.96498(3)	0.01086(12)
W16	0.30756(4)	0.04056(4)	0.05915(3)	0.00995(12)
W17	0.21015(4)	0.17840(4)	0.18781(2)	0.00920(12)
W18	0.20781(4)	0.40468(4)	0.18348(2)	0.00951(12)

W19	0.46984(4)	0.36680(4)	0.15199(2)	0.00860(12)
W20	0.56156(4)	0.23897(4)	0.03320(2)	0.00803(12)
W21	0.47198(4)	0.14057(4)	0.15558(2)	0.00852(12)
W22	0.39755(4)	0.13860(4)	0.93670(2)	0.00887(12)
W23	0.39616(4)	0.38306(4)	0.93192(2)	0.00875(12)
W24	0.30517(4)	0.50982(4)	0.05007(2)	0.00893(12)
La1	0.81348(7)	0.19928(6)	0.69716(4)	0.01447(18)
O01Y	0.6384(12)	0.2215(11)	0.2779(7)	0.041(4)
O01E	0.9653(10)	0.3244(9)	0.2658(5)	0.026(3)
O02X	0.7641(15)	0.2055(14)	0.1860(8)	0.061(5)
O02W	0.9250(12)	0.3481(13)	0.8577(11)	0.072(7)
O02T	0.6806(11)	0.3834(10)	0.3220(7)	0.038(3)
O02Q	0.2366(12)	0.2304(12)	0.7951(8)	0.048(4)
O02M	0.8556(10)	0.1545(9)	0.4270(6)	0.032(3)
O018	0.9717(10)	0.0851(9)	0.5190(5)	0.028(3)
O023	0.8551(10)	0.3192(10)	0.3720(5)	0.030(3)
O030	0.0995(11)	0.5116(11)	0.7938(6)	0.040(4)
O1	0.3668(7)	0.4588(7)	0.4281(4)	0.0094(19)
O2	0.2468(7)	0.3966(7)	0.3597(4)	0.0083(19)
O3	0.0922(7)	0.3170(6)	0.3632(4)	0.0078(19)
O4	0.0237(8)	0.2394(7)	0.4582(5)	0.012(2)
O5	0.0245(7)	0.4120(7)	0.4569(4)	0.010(2)
O7	0.1885(7)	0.2990(6)	0.4627(4)	0.0071(18)
O8	0.2999(7)	0.1974(6)	0.5295(4)	0.0074(19)
O9	0.5068(7)	0.1602(7)	0.5134(5)	0.013(2)
O10	0.5111(7)	0.3337(7)	0.3929(4)	0.010(2)
O11	0.5079(7)	0.1587(7)	0.3935(4)	0.0089(19)
O12	0.4041(9)	0.0815(7)	0.3069(5)	0.018(2)
O13	0.2441(7)	0.1857(7)	0.3613(4)	0.011(2)
O14	0.3892(8)	0.2667(7)	0.3237(4)	0.011(2)
O16	0.3580(8)	0.0813(7)	0.4305(5)	0.012(2)

O17	0.2108(7)	0.0679(6)	0.5693(4)	0.0064(18)
O18	0.0991(7)	0.2246(7)	0.5667(4)	0.0089(19)
O19	0.3345(7)	0.1320(6)	0.6381(4)	0.0077(19)
O20	0.2188(7)	0.2911(6)	0.6348(4)	0.0060(18)
O21	0.1512(8)	0.4667(7)	0.6765(5)	0.015(2)
O22	0.2205(7)	0.5179(7)	0.5664(4)	0.010(2)
O23	0.3401(7)	0.4100(7)	0.6353(4)	0.011(2)
O24	0.5424(8)	0.3985(8)	0.6255(5)	0.017(2)
O25	0.5103(7)	0.3303(7)	0.5120(4)	0.0087(19)
O26	0.4426(7)	0.2547(7)	0.6050(5)	0.010(2)
O27	0.4086(8)	0.4883(7)	0.5424(5)	0.012(2)
O28	0.1030(7)	0.3957(7)	0.5668(5)	0.010(2)
O29	0.3993(8)	0.0369(7)	0.5449(4)	0.012(2)
O30	0.1751(7)	0.1093(7)	0.4541(5)	0.012(2)
O31	0.2736(7)	0.9388(7)	0.4815(4)	0.010(2)
O32	0.5349(7)	0.0783(7)	0.6269(4)	0.011(2)
O33	0.1856(7)	0.4877(6)	0.4532(4)	0.0078(19)
O34	0.4108(7)	0.4488(7)	0.3052(4)	0.010(2)
O35	0.2910(7)	0.6257(7)	0.4783(4)	0.0089(19)
O36	0.1554(7)	0.2945(8)	0.9413(4)	0.013(2)
O37	0.0877(7)	0.2202(7)	0.0349(5)	0.012(2)
O38	0.1956(8)	0.0632(7)	0.0099(5)	0.015(2)
O39	0.1791(8)	0.4800(8)	0.2375(5)	0.018(2)
O40	0.2001(8)	0.2928(7)	0.2243(4)	0.011(2)
O42	0.5704(7)	0.1526(7)	0.0967(4)	0.010(2)
O43	0.5338(8)	0.0653(7)	0.2034(5)	0.014(2)
O44	0.5000(7)	0.2509(7)	0.1881(4)	0.010(2)
O45	0.3472(7)	0.1656(7)	0.1907(4)	0.009(2)
O46	0.4151(7)	0.0710(7)	0.1007(4)	0.0096(19)
O47	0.5007(7)	0.1632(7)	0.9865(4)	0.010(2)
O48	0.2964(7)	0.1930(7)	0.0227(4)	0.0083(19)

P2	0.3075(2)	0.2751(2)	0.05939(14)	0.0027(6)
O50	0.2659(7)	0.1408(7)	0.9148(4)	0.011(2)
O51	0.3834(8)	0.0385(7)	0.9864(5)	0.012(2)
O52	0.3811(7)	0.2630(7)	0.9167(5)	0.011(2)
O53	0.4559(8)	0.4120(7)	0.8702(4)	0.013(2)
O54	0.4988(7)	0.3326(7)	0.9828(4)	0.0091(19)
O55	0.2627(7)	0.4169(7)	0.9093(4)	0.011(2)
O56	0.3794(8)	0.4903(8)	0.9779(5)	0.018(2)
O57	0.4143(8)	0.4527(7)	0.0950(4)	0.012(2)
O58	0.2293(8)	0.4776(7)	0.1152(5)	0.015(2)
O59	0.3071(8)	0.6207(8)	0.0615(5)	0.018(2)
O60	0.1931(8)	0.5165(7)	0.0004(5)	0.015(2)
O61	0.4561(8)	0.0914(7)	0.8751(5)	0.016(2)
O62	0.5679(7)	0.3272(7)	0.0940(4)	0.0087(19)
O63	0.3461(7)	0.3780(6)	0.1872(4)	0.0081(19)
O64	0.0824(7)	0.3945(8)	0.1511(5)	0.015(2)
O65	0.0839(8)	0.2199(7)	0.1548(5)	0.012(2)
O66	0.1837(8)	0.1113(7)	0.2453(4)	0.014(2)
O67	0.2316(8)	0.0958(7)	0.1227(5)	0.011(2)
O68	0.0885(8)	0.3909(7)	0.0316(5)	0.015(2)
O69	0.0626(8)	0.4702(8)	0.9156(5)	0.017(2)
O70	0.7804(11)	0.2965(8)	0.7907(5)	0.030(3)
O71	0.6303(9)	0.2260(8)	0.7143(6)	0.026(3)
O72	0.7398(9)	0.3647(8)	0.6680(5)	0.022(3)
O201	0.3633(7)	0.2700(6)	0.4398(4)	0.0058(18)
O202	0.2948(7)	0.3601(6)	0.0190(4)	0.0067(18)
O203	0.6779(7)	0.2210(7)	0.0072(5)	0.014(2)
O204	0.2308(7)	0.2869(6)	0.1086(4)	0.0078(19)
O205	0.3038(7)	0.3613(6)	0.5282(5)	0.010(2)
O206	0.6609(7)	0.2221(7)	0.4480(4)	0.011(2)
O207	0.4085(7)	0.2622(6)	0.0877(4)	0.0066(18)

O208	0.0554(8)	0.1408(7)	0.3537(5)	0.015(2)
O209	0.9220(8)	0.3384(7)	0.5492(5)	0.014(2)
O210	0.1429(8)	0.1419(7)	0.6788(5)	0.014(2)
O211	0.5321(8)	0.4243(7)	0.1977(5)	0.014(2)
O212	0.0665(8)	0.1448(9)	0.9223(5)	0.022(3)
O213	0.3128(9)	0.9313(8)	0.0758(6)	0.022(3)
O214	0.0641(7)	0.5061(7)	0.3526(4)	0.011(2)
O215	0.7648(9)	0.2115(8)	0.5882(5)	0.022(3)
O216	0.7375(8)	0.0601(8)	0.6630(6)	0.023(3)
O217	0.9336(8)	0.3295(8)	0.0930(5)	0.019(2)
O218	0.7838(10)	0.0978(9)	0.7835(6)	0.030(3)
O219	0.9373(9)	0.0913(10)	0.6411(6)	0.029(3)
O220	0.3520(13)	0.7583(10)	0.1356(6)	0.041(4)
O221	0.9385(8)	0.3035(8)	0.6838(6)	0.022(3)
O222	0.9691(10)	0.1516(10)	0.7599(6)	0.032(3)
O223	0.9302(10)	0.6636(11)	0.2252(7)	0.040(4)
O224	0.4089(10)	0.9258(9)	0.2253(6)	0.027(3)
O225	0.9297(12)	0.1554(13)	0.2405(8)	0.052(4)
O226	0.7621(12)	0.0487(10)	0.1156(7)	0.043(4)
O227	0.7399(10)	0.3702(10)	0.1295(7)	0.034(3)
O228	0.6970(12)	0.0423(12)	0.3112(8)	0.054(5)
O229	0.8886(16)	0.0548(11)	0.3298(7)	0.055(5)
O230	0.423(2)	0.6077(18)	0.2314(12)	0.091(7)
O232	0.0730(19)	0.6697(18)	0.0476(11)	0.031(9)
O233	0.768(2)	0.9407(16)	0.2086(9)	0.090(8)
O234	0.7166(16)	0.4877(15)	0.2245(10)	0.069(6)
O235	0.0379(19)	0.0039(18)	0.1697(11)	0.041(9)
O236	0.622(2)	0.7125(18)	0.2540(12)	0.091(7)
O237	0.947(2)	0.056(2)	0.0573(14)	0.044(11)
O238	0.127(3)	0.789(3)	0.0246(18)	0.17(2)

Table TS8. Bond lengths (Å) for LaPW_LT12_221118.

W1-O35	1.693(10)	W1-O1	1.885(10)
W1-O33	1.912(9)	W1-O27	1.912(11)
W1-O22	1.942(10)	W1-O205	2.429(10)
P1-O7	1.522(9)	P1-O201	1.524(9)
P1-O8	1.525(10)	P1-O205	1.530(10)
W2-O34	1.696(10)	W2-O2	1.889(10)
W2-O1	1.910(10)	W2-O14	1.922(10)
W2-O10	1.953(10)	W2-O201	2.443(9)
W3-O214	1.698(10)	W3-O33	1.883(9)
W3-O2	1.919(10)	W3-O5	1.920(10)
W3-O3	1.931(10)	W3-O7	2.439(10)
W4-O208	1.696(10)	W4-O13	1.908(10)
W4-O30	1.915(10)	W4-O3	1.917(10)
W4-O4	1.920(10)	W4-O7	2.427(9)
W5-O209	1.692(11)	W5-O28	1.898(10)
W5-O5	1.907(10)	W5-O4	1.919(10)
W5-O18	1.931(10)	W5-O7	2.463(10)
W6-O32	1.708(10)	W6-O9	1.871(11)
W6-O29	1.901(11)	W6-O26	1.909(10)
W6-O19	1.939(10)	W6-O8	2.426(9)
W7-O206	1.699(10)	W7-O10	1.890(10)
W7-O11	1.912(10)	W7-O25	1.923(10)
W7-O9	1.936(11)	W7-O201	2.435(9)
W8-O31	1.710(10)	W8-O16	1.891(11)
W8-O30	1.912(10)	W8-O17	1.929(9)
W8-O29	1.943(11)	W8-O8	2.449(10)
W9-O210	1.692(11)	W9-O18	1.892(10)
W9-O20	1.898(9)	W9-O17	1.915(9)
W9-O19	1.936(10)	W9-O8	2.440(10)
W10-O21	1.701(10)	W10-O28	1.896(10)

W10-O22	1.907(11)	W10-O23	1.911(10)
W10-O20	1.915(9)	W10-O205	2.440(10)
W11-O24	1.710(11)	W11-O25	1.882(10)
W11-O26	1.904(11)	W11-O23	1.923(10)
W11-O27	1.926(11)	W11-O205	2.430(10)
W12-O12	1.699(10)	W12-O13	1.887(10)
W12-O14	1.908(10)	W12-O16	1.914(11)
W12-O11	1.931(10)	W12-O201	2.438(9)
W13-O217	1.707(12)	W13-O64	1.901(11)
W13-O37	1.908(10)	W13-O68	1.914(11)
W13-O65	1.928(11)	W13-O204	2.432(10)
W14-O69	1.717(11)	W14-O68	1.892(11)
W14-O55	1.897(10)	W14-O36	1.905(11)
W14-O60	1.927(11)	W14-O202	2.425(9)
W15-O212	1.685(12)	W15-O36	1.904(11)
W15-O37	1.904(10)	W15-O50	1.914(10)
W15-O38	1.941(12)	W15-O48	2.444(10)
W16-O213	1.680(12)	W16-O67	1.890(10)
W16-O46	1.903(10)	W16-O38	1.911(11)
W16-O51	1.931(11)	W16-O48	2.417(10)
W17-O66	1.699(11)	W17-O45	1.901(10)
W17-O40	1.912(10)	W17-O67	1.928(10)
W17-O65	1.933(11)	W17-O204	2.441(10)
W18-O39	1.678(11)	W18-O63	1.917(10)
W18-O58	1.918(11)	W18-O40	1.928(10)
W18-O64	1.940(10)	W18-O204	2.450(9)
W19-O211	1.692(10)	W19-O63	1.879(10)
W19-O57	1.890(10)	W19-O44	1.906(10)
W19-O62	1.910(10)	W19-O207	2.422(9)
W20-O203	1.705(10)	W20-O47	1.874(10)
W20-O54	1.905(10)	W20-O42	1.905(10)

W20-O62	1.937(10)	W20-O207	2.431(10)
W21-O43	1.692(10)	W21-O45	1.892(10)
W21-O46	1.895(10)	W21-O42	1.922(10)
W21-O44	1.932(10)	W21-O207	2.430(9)
W22-O61	1.704(10)	W22-O51	1.898(11)
W22-O52	1.907(11)	W22-O50	1.910(10)
W22-O47	1.923(10)	W22-O48	2.450(10)
W23-O53	1.692(10)	W23-O52	1.900(10)
W23-O54	1.901(10)	W23-O56	1.918(12)
W23-O55	1.930(10)	W23-O202	2.447(10)
W24-O59	1.707(11)	W24-O58	1.894(11)
W24-O56	1.912(10)	W24-O57	1.920(10)
W24-O60	1.925(11)	W24-O202	2.410(9)
La1-O218	2.519(14)	La1-O219	2.525(13)
La1-O215	2.537(11)	La1-O221	2.548(12)
La1-O71	2.558(13)	La1-O70	2.575(11)
La1-O222	2.607(13)	La1-O72	2.628(12)
La1-O216	2.636(12)	O48-P2	1.531(10)
P2-O204	1.521(10)	P2-O207	1.541(10)
P2-O202	1.548(10)		

Table TS9. Bond angles (°) for LaPW_LT12_221118.

O35-W1-O1	104.3(4)	O35-W1-O33	102.4(4)
O1-W1-O33	84.9(4)	O35-W1-O27	102.9(5)
O1-W1-O27	90.3(4)	O33-W1-O27	154.7(4)
O35-W1-O22	100.9(4)	O1-W1-O22	154.7(4)
O33-W1-O22	86.9(4)	O27-W1-O22	86.9(4)
O35-W1-O205	172.6(4)	O1-W1-O205	82.3(4)
O33-W1-O205	81.5(4)	O27-W1-O205	73.3(4)
O22-W1-O205	72.8(4)	O7-P1-O201	108.1(5)
O7-P1-O8	110.3(5)	O201-P1-O8	109.7(5)

O7-P1-O205	110.0(5)	O201-P1-O205	109.4(5)
O8-P1-O205	109.2(5)	O34-W2-O2	102.7(5)
O34-W2-O1	102.3(5)	O2-W2-O1	85.7(4)
O34-W2-O14	103.4(5)	O2-W2-O14	90.0(4)
O1-W2-O14	154.2(4)	O34-W2-O10	101.7(5)
O2-W2-O10	155.5(4)	O1-W2-O10	87.1(4)
O14-W2-O10	86.3(4)	O34-W2-O201	172.7(4)
O2-W2-O201	83.6(4)	O1-W2-O201	81.6(4)
O14-W2-O201	72.7(4)	O10-W2-O201	72.2(4)
O214-W3-O33	103.9(5)	O214-W3-O2	102.5(4)
O33-W3-O2	85.8(4)	O214-W3-O5	101.6(5)
O33-W3-O5	89.9(4)	O2-W3-O5	155.8(4)
O214-W3-O3	102.6(5)	O33-W3-O3	153.5(4)
O2-W3-O3	86.5(4)	O5-W3-O3	86.8(4)
O214-W3-O7	172.8(4)	O33-W3-O7	81.6(4)
O2-W3-O7	82.3(4)	O5-W3-O7	73.5(4)
O3-W3-O7	72.2(4)	O208-W4-O13	103.8(5)
O208-W4-O30	102.0(5)	O13-W4-O30	86.0(4)
O208-W4-O3	101.8(5)	O13-W4-O3	88.6(4)
O30-W4-O3	156.2(4)	O208-W4-O4	100.8(5)
O13-W4-O4	155.3(4)	O30-W4-O4	87.7(4)
O3-W4-O4	87.5(4)	O208-W4-O7	171.5(4)
O13-W4-O7	82.8(4)	O30-W4-O7	83.6(4)
O3-W4-O7	72.7(4)	O4-W4-O7	72.8(4)
O209-W5-O28	104.5(5)	O209-W5-O5	102.6(5)
O28-W5-O5	90.4(4)	O209-W5-O4	101.0(5)
O28-W5-O4	154.4(4)	O5-W5-O4	86.3(4)
O209-W5-O18	101.8(5)	O28-W5-O18	85.5(4)
O5-W5-O18	155.4(4)	O4-W5-O18	87.1(4)
O209-W5-O7	171.7(4)	O28-W5-O7	82.8(4)
O5-W5-O7	73.1(4)	O4-W5-O7	71.9(4)

O18-W5-O7	82.3(4)	O32-W6-O9	103.4(5)
O32-W6-O29	102.1(5)	O9-W6-O29	89.6(4)
O32-W6-O26	102.1(4)	O9-W6-O26	86.5(5)
O29-W6-O26	155.8(4)	O32-W6-O19	100.4(4)
O9-W6-O19	156.2(4)	O29-W6-O19	86.3(4)
O26-W6-O19	87.7(4)	O32-W6-O8	172.2(4)
O9-W6-O8	83.2(4)	O29-W6-O8	73.5(4)
O26-W6-O8	82.4(4)	O19-W6-O8	73.2(4)
O206-W7-O10	100.7(5)	O206-W7-O11	100.8(5)
O10-W7-O11	88.6(4)	O206-W7-O25	103.3(5)
O10-W7-O25	88.7(4)	O11-W7-O25	155.8(4)
O206-W7-O9	103.7(5)	O10-W7-O9	155.6(4)
O11-W7-O9	88.5(4)	O25-W7-O9	84.1(4)
O206-W7-O201	171.6(4)	O10-W7-O201	73.3(4)
O11-W7-O201	73.5(4)	O25-W7-O201	82.7(4)
O9-W7-O201	82.6(4)	O31-W8-O16	103.7(5)
O31-W8-O30	100.6(5)	O16-W8-O30	85.4(5)
O31-W8-O17	101.8(4)	O16-W8-O17	154.4(4)
O30-W8-O17	88.4(4)	O31-W8-O29	103.8(5)
O16-W8-O29	88.8(4)	O30-W8-O29	155.6(4)
O17-W8-O29	86.7(4)	O31-W8-O8	173.1(4)
O16-W8-O8	82.1(4)	O30-W8-O8	83.5(4)
O17-W8-O8	72.5(4)	O29-W8-O8	72.3(4)
O210-W9-O18	103.4(5)	O210-W9-O20	102.5(5)
O18-W9-O20	87.0(4)	O210-W9-O17	101.9(5)
O18-W9-O17	87.3(4)	O20-W9-O17	155.6(4)
O210-W9-O19	101.1(5)	O18-W9-O19	155.5(4)
O20-W9-O19	88.8(4)	O17-W9-O19	86.6(4)
O210-W9-O8	172.0(4)	O18-W9-O8	82.6(4)
O20-W9-O8	82.8(4)	O17-W9-O8	72.9(4)
O19-W9-O8	72.9(4)	O21-W10-O28	103.6(5)

O21-W10-O22	100.5(5)	O28-W10-O22	88.9(4)
O21-W10-O23	101.4(5)	O28-W10-O23	154.9(4)
O22-W10-O23	87.5(4)	O21-W10-O20	103.6(5)
O28-W10-O20	84.9(4)	O22-W10-O20	155.8(4)
O23-W10-O20	88.3(4)	O21-W10-O205	170.9(5)
O28-W10-O205	83.0(4)	O22-W10-O205	73.1(4)
O23-W10-O205	72.2(4)	O20-W10-O205	82.9(4)
O24-W11-O25	103.7(5)	O24-W11-O26	102.2(5)
O25-W11-O26	86.7(4)	O24-W11-O23	101.5(5)
O25-W11-O23	154.8(4)	O26-W11-O23	87.5(4)
O24-W11-O27	102.2(5)	O25-W11-O27	89.4(4)
O26-W11-O27	155.6(4)	O23-W11-O27	85.8(4)
O24-W11-O205	172.2(4)	O25-W11-O205	82.6(4)
O26-W11-O205	82.6(4)	O23-W11-O205	72.3(4)
O27-W11-O205	73.0(4)	O12-W12-O13	102.8(5)
O12-W12-O14	102.2(5)	O13-W12-O14	88.8(5)
O12-W12-O16	103.1(5)	O13-W12-O16	85.2(4)
O14-W12-O16	154.8(4)	O12-W12-O11	101.6(5)
O13-W12-O11	155.6(4)	O14-W12-O11	87.1(4)
O16-W12-O11	88.3(4)	O12-W12-O201	172.8(5)
O13-W12-O201	82.7(4)	O14-W12-O201	73.0(4)
O16-W12-O201	82.0(4)	O11-W12-O201	73.1(4)
O217-W13-O64	101.0(5)	O217-W13-O37	102.5(5)
O64-W13-O37	156.5(4)	O217-W13-O68	102.3(5)
O64-W13-O68	89.1(5)	O37-W13-O68	85.1(5)
O217-W13-O65	101.9(5)	O64-W13-O65	87.1(5)
O37-W13-O65	88.8(4)	O68-W13-O65	155.7(5)
O217-W13-O204	172.1(4)	O64-W13-O204	72.9(4)
O37-W13-O204	83.8(4)	O68-W13-O204	82.9(4)
O65-W13-O204	73.1(4)	O69-W14-O68	103.9(5)
O69-W14-O55	101.2(5)	O68-W14-O55	155.0(4)

O69-W14-O36	102.2(5)	O68-W14-O36	86.0(5)
O55-W14-O36	88.3(4)	O69-W14-O60	101.5(5)
O68-W14-O60	88.1(5)	O55-W14-O60	87.3(4)
O36-W14-O60	156.3(4)	O69-W14-O202	171.3(5)
O68-W14-O202	82.1(4)	O55-W14-O202	73.1(4)
O36-W14-O202	84.3(4)	O60-W14-O202	72.1(4)
O212-W15-O36	102.2(5)	O212-W15-O37	103.7(5)
O36-W15-O37	86.1(4)	O212-W15-O50	101.3(5)
O36-W15-O50	88.1(4)	O37-W15-O50	155.0(4)
O212-W15-O38	101.8(6)	O36-W15-O38	156.0(4)
O37-W15-O38	88.0(4)	O50-W15-O38	87.5(4)
O212-W15-O48	171.5(5)	O36-W15-O48	84.0(4)
O37-W15-O48	82.6(4)	O50-W15-O48	72.6(4)
O38-W15-O48	72.3(4)	O213-W16-O67	102.4(5)
O213-W16-O46	102.9(5)	O67-W16-O46	85.7(4)
O213-W16-O38	101.7(6)	O67-W16-O38	88.4(4)
O46-W16-O38	155.4(4)	O213-W16-O51	102.6(5)
O67-W16-O51	155.0(4)	O46-W16-O51	88.3(4)
O38-W16-O51	87.0(4)	O213-W16-O48	173.0(5)
O67-W16-O48	82.7(4)	O46-W16-O48	82.2(4)
O38-W16-O48	73.4(4)	O51-W16-O48	72.5(4)
O66-W17-O45	102.6(5)	O66-W17-O40	102.7(5)
O45-W17-O40	90.0(4)	O66-W17-O67	102.0(5)
O45-W17-O67	85.3(4)	O40-W17-O67	155.3(4)
O66-W17-O65	102.0(5)	O45-W17-O65	155.3(4)
O40-W17-O65	86.9(4)	O67-W17-O65	87.4(4)
O66-W17-O204	173.2(4)	O45-W17-O204	82.8(4)
O40-W17-O204	72.9(4)	O67-W17-O204	82.4(4)
O65-W17-O204	72.8(4)	O39-W18-O63	103.3(5)
O39-W18-O58	102.9(5)	O63-W18-O58	85.3(4)
O39-W18-O40	102.9(5)	O63-W18-O40	89.1(4)

O58-W18-O40	154.2(4)	O39-W18-O64	102.4(5)
O63-W18-O64	154.3(4)	O58-W18-O64	88.2(5)
O40-W18-O64	86.0(5)	O39-W18-O204	172.6(5)
O63-W18-O204	82.6(4)	O58-W18-O204	81.9(4)
O40-W18-O204	72.4(4)	O64-W18-O204	71.9(4)
O211-W19-O63	104.1(5)	O211-W19-O57	104.6(5)
O63-W19-O57	86.4(4)	O211-W19-O44	99.2(5)
O63-W19-O44	89.0(4)	O57-W19-O44	156.1(4)
O211-W19-O62	100.2(5)	O63-W19-O62	155.7(4)
O57-W19-O62	87.8(4)	O44-W19-O62	86.8(4)
O211-W19-O207	168.7(4)	O63-W19-O207	83.7(4)
O57-W19-O207	83.8(4)	O44-W19-O207	72.5(4)
O62-W19-O207	72.2(4)	O203-W20-O47	103.1(5)
O203-W20-O54	104.0(5)	O47-W20-O54	85.3(4)
O203-W20-O42	100.2(5)	O47-W20-O42	89.4(4)
O54-W20-O42	155.8(4)	O203-W20-O62	101.6(5)
O47-W20-O62	155.3(4)	O54-W20-O62	88.4(4)
O42-W20-O62	86.7(4)	O203-W20-O207	169.9(4)
O47-W20-O207	84.0(4)	O54-W20-O207	83.6(4)
O42-W20-O207	72.4(4)	O62-W20-O207	71.5(4)
O43-W21-O45	103.8(5)	O43-W21-O46	104.9(5)
O45-W21-O46	85.7(4)	O43-W21-O42	100.9(5)
O45-W21-O42	155.3(4)	O46-W21-O42	88.6(4)
O43-W21-O44	100.5(5)	O45-W21-O44	88.5(4)
O46-W21-O44	154.6(4)	O42-W21-O44	86.4(4)
O43-W21-O207	169.7(4)	O45-W21-O207	83.3(4)
O46-W21-O207	82.9(4)	O42-W21-O207	72.2(4)
O44-W21-O207	71.9(4)	O61-W22-O51	103.4(5)
O61-W22-O52	102.2(5)	O51-W22-O52	154.4(4)
O61-W22-O50	100.8(5)	O51-W22-O50	86.8(4)
O52-W22-O50	87.6(4)	O61-W22-O47	103.6(5)

O51-W22-O47	89.6(4)	O52-W22-O47	85.1(4)
O50-W22-O47	155.4(4)	O61-W22-O48	172.0(5)
O51-W22-O48	72.2(4)	O52-W22-O48	82.3(4)
O50-W22-O48	72.6(4)	O47-W22-O48	83.2(4)
O53-W23-O52	102.8(5)	O53-W23-O54	102.4(5)
O52-W23-O54	85.8(4)	O53-W23-O56	102.9(5)
O52-W23-O56	154.2(5)	O54-W23-O56	89.5(4)
O53-W23-O55	102.2(5)	O52-W23-O55	87.2(4)
O54-W23-O55	155.3(4)	O56-W23-O55	86.6(4)
O53-W23-O202	172.3(4)	O52-W23-O202	82.2(4)
O54-W23-O202	83.6(4)	O56-W23-O202	72.1(4)
O55-W23-O202	72.0(4)	O59-W24-O58	102.9(5)
O59-W24-O56	101.6(5)	O58-W24-O56	155.5(5)
O59-W24-O57	102.7(5)	O58-W24-O57	85.7(5)
O56-W24-O57	89.9(4)	O59-W24-O60	100.7(5)
O58-W24-O60	88.4(5)	O56-W24-O60	86.2(5)
O57-W24-O60	156.6(5)	O59-W24-O202	171.4(4)
O58-W24-O202	82.5(4)	O56-W24-O202	73.1(4)
O57-W24-O202	84.2(4)	O60-W24-O202	72.5(4)
O218-La1-O219	98.3(4)	O218-La1-O215	134.6(4)
O219-La1-O215	73.4(4)	O218-La1-O221	130.3(4)
O219-La1-O221	83.3(4)	O215-La1-O221	93.7(4)
O218-La1-O71	74.5(4)	O219-La1-O71	137.9(4)
O215-La1-O71	82.6(4)	O221-La1-O71	133.3(4)
O218-La1-O70	72.4(4)	O219-La1-O70	145.4(4)
O215-La1-O70	136.7(4)	O221-La1-O70	78.8(4)
O71-La1-O70	73.1(4)	O218-La1-O222	68.4(4)
O219-La1-O222	68.6(4)	O215-La1-O222	138.5(4)
O221-La1-O222	66.3(4)	O71-La1-O222	137.9(4)
O70-La1-O222	77.1(5)	O218-La1-O72	133.0(4)
O219-La1-O72	128.7(4)	O215-La1-O72	69.0(4)

O221-La1-O72	65.9(4)	O71-La1-O72	69.5(4)
O70-La1-O72	69.0(4)	O222-La1-O72	125.3(4)
O218-La1-O216	67.7(4)	O219-La1-O216	69.6(4)
O215-La1-O216	67.7(4)	O221-La1-O216	150.3(4)
O71-La1-O216	69.4(4)	O70-La1-O216	130.7(4)
O222-La1-O216	112.0(4)	O72-La1-O216	122.7(4)
W1-O1-W2	153.5(6)	W2-O2-W3	151.2(5)
W4-O3-W3	125.5(5)	W5-O4-W4	126.3(6)
W5-O5-W3	125.5(5)	P1-O7-W4	126.6(5)
P1-O7-W3	126.9(5)	W4-O7-W3	89.3(3)
P1-O7-W5	125.0(5)	W4-O7-W5	88.9(3)
W3-O7-W5	87.9(3)	P1-O8-W6	126.2(5)
P1-O8-W9	126.0(5)	W6-O8-W9	89.5(3)
P1-O8-W8	125.6(5)	W6-O8-W8	88.9(3)
W9-O8-W8	88.8(3)	W6-O9-W7	153.0(6)
W7-O10-W2	125.5(5)	W7-O11-W12	124.6(5)
W12-O13-W4	152.3(6)	W12-O14-W2	125.7(5)
W8-O16-W12	153.6(6)	W9-O17-W8	125.7(5)
W9-O18-W5	150.8(6)	W9-O19-W6	124.4(5)
W9-O20-W10	151.7(5)	W10-O22-W1	125.0(5)
W10-O23-W11	126.2(5)	W11-O25-W7	152.9(6)
W11-O26-W6	150.4(6)	W1-O27-W11	124.8(6)
W10-O28-W5	153.9(6)	W6-O29-W8	125.3(5)
W8-O30-W4	150.9(6)	W3-O33-W1	153.7(6)
W14-O36-W15	150.9(6)	W15-O37-W13	151.9(6)
W16-O38-W15	125.0(6)	W17-O40-W18	125.9(5)
W20-O42-W21	126.2(5)	W19-O44-W21	126.0(5)
W21-O45-W17	152.6(6)	W21-O46-W16	152.6(6)
W20-O47-W22	152.7(6)	P2-O48-W16	126.7(5)
P2-O48-W15	126.0(5)	W16-O48-W15	89.3(3)
P2-O48-W22	125.4(5)	W16-O48-W22	89.1(3)

W15-O48-W22	88.4(3)	O204-P2-O48	109.3(6)
O204-P2-O207	109.3(5)	O48-P2-O207	109.8(5)
O204-P2-O202	109.0(5)	O48-P2-O202	110.2(5)
O207-P2-O202	109.2(5)	W15-O50-W22	126.4(5)
W22-O51-W16	126.2(6)	W23-O52-W22	152.5(6)
W23-O54-W20	151.9(6)	W14-O55-W23	126.0(5)
W24-O56-W23	125.7(7)	W19-O57-W24	150.9(6)
W24-O58-W18	152.6(6)	W24-O60-W14	125.2(6)
W19-O62-W20	126.2(5)	W19-O63-W18	152.1(6)
W13-O64-W18	126.1(6)	W13-O65-W17	124.8(5)
W16-O67-W17	151.9(6)	W14-O68-W13	152.6(6)
P1-O201-W7	126.1(5)	P1-O201-W12	126.5(5)
W7-O201-W12	88.6(3)	P1-O201-W2	126.0(5)
W7-O201-W2	88.9(3)	W12-O201-W2	88.6(3)
P2-O202-W24	126.2(5)	P2-O202-W14	126.0(5)
W24-O202-W14	90.1(3)	P2-O202-W23	125.0(5)
W24-O202-W23	89.1(3)	W14-O202-W23	88.9(3)
P2-O204-W13	125.5(5)	P2-O204-W17	126.1(5)
W13-O204-W17	89.2(3)	P2-O204-W18	126.4(5)
W13-O204-W18	89.0(3)	W17-O204-W18	88.8(3)
P1-O205-W1	126.1(6)	P1-O205-W11	126.0(5)
W1-O205-W11	88.9(3)	P1-O205-W10	125.7(6)
W1-O205-W10	89.0(3)	W11-O205-W10	89.2(3)
P2-O207-W19	126.0(5)	P2-O207-W21	125.1(5)
W19-O207-W21	89.6(3)	P2-O207-W20	125.6(5)
W19-O207-W20	90.0(3)	W21-O207-W20	89.2(3)
